# Sex-specific differences in immunogenomic features of response to immune checkpoint blockade

**DOI:** 10.3389/fonc.2022.945798

**Published:** 2022-08-03

**Authors:** Susan C. Scott, Xiaoshan M. Shao, Noushin Niknafs, Archana Balan, Gavin Pereira, Kristen A. Marrone, Vincent K. Lam, Joseph C. Murray, Josephine L. Feliciano, Benjamin P. Levy, David S. Ettinger, Christine L. Hann, Julie R. Brahmer, Patrick M. Forde, Rachel Karchin, Jarushka Naidoo, Valsamo Anagnostou

**Affiliations:** ^1^ The Sidney Kimmel Comprehensive Cancer Center, Johns Hopkins University School of Medicine, Baltimore, MD, United States; ^2^ Department of Biomedical Engineering, Johns Hopkins University, Baltimore, MD, United States; ^3^ Institute for Computational Medicine, Johns Hopkins University, Baltimore, MD, United States; ^4^ Department of Oncology, RCSI University of Medicine and Health Sciences, Dublin, Ireland; ^5^ Department of Oncology, Beaumont Hospital, Dublin, Ireland

**Keywords:** lung cancer, immunotherapy, cancer genomics, sex dimorphism, HLA zygosity

## Abstract

**Introduction:**

The magnitude of response to immune checkpoint inhibitor (ICI) therapy may be sex-dependent, as females have lower response rates and decreased survival after ICI monotherapy. The mechanisms underlying this sex dimorphism in ICI response are unknown, and may be related to sex-driven differences in the immunogenomic landscape of tumors that shape anti-tumor immune responses in the context of therapy.

**Methods:**

To investigate the association of immunogenic mutations with HLA haplotypes, we leveraged whole exome sequence data and HLA genotypes from 482 non-small cell lung cancer (NSCLC) tumors from The Cancer Genome Atlas (TCGA). To explore sex-specific genomic features linked with ICI response, we analyzed whole exome sequence data from patients with NSCLC treated with ICI. Tumor mutational burden (TMB), HLA class I and II restricted immunogenic missense mutation (IMM) load, and mutational smoking signature were defined for each tumor. IMM load was combined with HLA class I and II haplotypes and correlated with therapeutic response and survival following ICI treatment. We examined rates of durable clinical benefit (DCB) for at least six months from ICI treatment initiation. Findings were validated utilizing whole exome sequence data from an independent cohort of ICI treated NSCLC.

**Results:**

Analysis of whole exome sequence data from NSCLC tumors of females and males revealed that germline HLA class II diversity (≥9 unique HLA alleles) was associated with higher tumor class II IMM load in females (p=0.01) and not in males (p=0.64). Similarly, in tumors of female patients, somatic HLA class II loss of heterozygosity was associated with increased IMM load (p=0.01) while this association was not observed in tumors in males (p=0.20). In females, TMB (p=0.005), class I IMM load (p=0.005), class II IMM load (p=0.004), and mutational smoking signature (p<0.001) were significantly higher in tumors responding to ICI as compared to non-responding tumors. In contrast, among males, there was no significant association between DCB and any of these features. When IMM was considered in the context of HLA zygosity, high MHC-II restricted IMM load and high HLA class II diversity was significantly associated with overall survival in males (p=0.017).

**Conclusions:**

Inherent sex-driven differences in immune surveillance affect the immunogenomic determinants of response to ICI and likely mediate the dimorphic outcomes with ICI therapy. Deeper understanding of the selective pressures and mechanisms of immune escape in tumors in males and females can inform patient selection strategies and can be utilized to further hone immunotherapy approaches in cancer.

## Introduction

Immune checkpoint inhibitor (ICI) therapies improve outcomes and are approved for use in multiple treatment settings across tumor types with a rapidly expanding repertoire of agents, applications, and combination strategies (1). However, only a fraction of patients benefit from treatment and an even smaller fraction achieve long-term disease control. Despite hundreds of clinical trials of ICI therapies in cancer, there is paucity of robust biomarker models to predict response (2, 3). Furthermore, ICIs are not without risk, and improved predictive biomarkers will help to limit exposure in patients unlikely to benefit.

Several recent meta-analyses of immunotherapy clinical trials have suggested a decreased magnitude of benefit for ICI monotherapy in female patients as compared to male patients across multiple cancer types, including non-small cell lung cancer (NSCLC) (4–9). A meta-analysis by Conforti *et al.* included six randomized trials with single- or dual-agent PD-1/PD-L1 inhibitors in NSCLC, including 3482 patients, and reported a greater magnitude of benefit in males with a pooled hazard ratio (HR) of 0.72 (95% CI 0.61-0.86) compared to a lack of significant benefit in females with a pooled HR of 0.89 (95% CI (0.71-1.11) (4). In all trials the HR for death was lower for males than females, and in three NSCLC trials (10–12) the HR demonstrated a significant benefit only for male and not for female patients. Interestingly, subsequent analysis has demonstrated that females have significantly increased magnitude of benefit from combinations of chemo-immunotherapy (13), and females experience higher rates of immune-related adverse events after immunotherapy for NSCLC and melanoma, as compared to males (14, 15). Together, the data highlight the significant yet incompletely understood impact of sex on outcomes following ICI therapy in multiple tumor types and treatment settings. These findings have spurred interest in investigating the underlying mechanisms potentially mediating this sex dimorphism in therapeutic response in the context of immunotherapy.

Inherent differences in the male and female immune response are likely central to the observed sex dimorphism in ICI response. Females demonstrate increased humoral and cell-mediated responses to antigenic stimulation, vaccination, and infection, and there exists a strong female predominance of autoimmune diseases (16, 17). Females demonstrate increased CD8+ T lymphocyte activity and IFNγ production as compared to males, and tumors in females demonstrate enrichment of nearly all T cell subpopulations in the tumor microenvironment (18, 19). Furthermore, estrogen signaling has been demonstrated to modulate the tumor immune microenvironment, tumor antigen presentation, immune checkpoint expression, and intratumoral lymphocyte infiltration (20, 21). Given these well-established differences in immune responses of males and females, we hypothesized that there are sex-based differences in tumor-intrinsic features that have been shaped by the immune system during tumor evolution and immunoediting, which are reflected in differential clinical outcomes following ICI therapy.

The therapeutic efficacy of ICIs relies on augmentation of the anti-tumor immune response, mediated by presentation of tumor-specific neoantigens primarily by major histocompatibility complex (MHC) class I proteins, along with multiple co-stimulatory signals, and resultant CD8+ effector cell activation. We recently developed MHCnuggets, a deep neural network method to predict peptide-MHC binding that incorporates HLA genotype and whole exome sequencing to quantify immunogenic missense mutations (IMM) (22). Utilizing these approaches, we have subsequently shown that tumors with high HLA class II restricted IMM loads are more likely to regress with immune checkpoint blockade (23). Considering IMM load in the context of HLA class I and II zygosity is important, as decreased HLA genetic variation may lead to immune escape and suboptimal responses to ICI therapy (24, 25).

Functional differences in anti-tumor immune activity between males and females likely contribute to the differential response to ICI therapy and may be driven by tumor intrinsic and host-related features. Here, we first evaluated the background association between immunogenic mutation load and HLA zygosity in a sex-dependent manner in NSCLC tumors from TCGA. To explore sex-specific genomic features linked with ICI response, we analyzed whole exome sequence data of two independent cohorts of patients with NSCLC treated with ICIs and assessed differences in the MHC I and II-restricted immunogenic mutation repertoire combined with the germline and somatic HLA class I and II zygosity. Our findings highlight sex-specific differences in immunogenomic determinants of response to ICI that may impact clinical decision making.

## Methods

### Cohort characteristics

The primary NSCLC cohort consisted of 89 patients treated with ICI therapy at Johns Hopkins Sidney Kimmel Cancer Center and the Nederlands Kanker Instituut; whole exome sequence and clinical metadata were retrieved from the original publication (25). In addition to sequence annotation of activating *EGFR* mutations, review of clinical next generation sequencing data was performed to identify *ALK, ROS1*, and *RET* rearrangements. *ALK* rearrangement status was not available for 7 of 89 tumors, *ROS1* and *RET* rearrangement status was not available for 13 tumors, including 2 in patients with no history of tobacco exposure (1 male and 1 female). Whole exome sequence data from a published cohort of 34 NSCLC patients treated with PD-1 blockade (NSCLC validation cohort) were obtained and analyzed to validate key findings from the primary NSCLC cohort (26). Driver gene fusion analyses were not available for this cohort. Genomic and demographic information of 286 lung adenocarcinoma (LUAD) and 196 lung squamous cell carcinoma (LUSC) samples from The Cancer Genome Atlas (TCGA) were retrieved from the NCI Genomic Data Commons (https://gdc.cancer.gov/about-data/publications/mc3-2017). Clinical annotations of tumors and structural variants including gene fusions were accessed using the TCGA clinical data resource (27).

### Assessment of clinical response following ICI therapy

All patients in each cohort were treated with anti-PD-1 or anti-PD-L1 therapy alone or in combination with anti-CTLA-4 therapy or chemotherapy. Given the challenges with conventional radiologic response assessments that may underestimate the unique patterns and timing of response to immune targeted therapies, we defined response as durable clinical benefit if complete response, partial response, or stable disease was achieved with a duration of >6 months. Responding and non-responding tumors, therefore refer to patients attaining durable clinical benefit (DCB) and non-durable clinical benefit (NDB), respectively. Overall survival was used to determine long-term outcome for the primary NSCLC cohort. Progression free survival only was available for the NSCLC validation cohort.

### Somatic mutation extraction and identification of putative immunogenic mutations

Missense somatic mutation calls for both primary and validation cohorts were extracted from the original publications (25, 28). Missense somatic mutation calls for the TCGA samples were obtained from Multi-Center Mutation Calling in Multiple Cancers (MC3; https://gdc.cancer.gov/about-data/publications/mc3-2017). The burdens of immunogenic mutations (IMM) were computed as previously described (23). In brief, using *varcode* (https://github.com/openvax/varcode), silent and nonsense mutations were filtered out of each patient’s mutation profiles, and mutant peptide sequences surrounding the affected amino acid for all missense mutations were extracted. Windowing around the affected amino acid, 8-11mers were extracted for HLA class I analyses, and 12-20mers, HLA class II analyses. Next, we employed MHCnuggets (22) to obtain the ranks of the binding affinities of all the mutation containing peptides against the respective HLA class I and II haplotypes of the patients (23). Each candidate peptide’s predicted MHC binding affinities were compared against the MHC binding affinities of a list of 100,000 human proteome peptides (23). Using a rank threshold of 0.01, we considered all the epitopes with predicted binding affinity over this rank immunogenic neoantigens. A putative IMM was thus defined as a missense mutation that contain as least one predicted mutation-associated neoantigens (MANA) fulfilling these criteria.

### Mutational signatures

The contribution of smoking-related mutational signatures in the mutational spectra of NSCLC tumors in the primary and validation cohorts were extracted from the original publication (25). In brief, the deconstructSigs R package was utilized (29) to calculate the contribution of COSMIC smoking signature 4 from all the coding point mutations in their trinucleotide context (30).

### HLA genotyping

HLA class I and class II germline genotypes for both primary and validation cohorts were identified as previously described (23, 25). In brief, using whole exome sequencing, each samples’ HLA class I germline haplotype (HLA-A, HLA-B, and HLA-C) were identified with OptiType (31). For HLA class II haplotypes, an ensemble approach utilizing SOAP-HLA (32) and xHLA (33) was employed such that xHLA was used to determine HLA-DPB1, HLA-DQB1 and HLA-DRB1 haplotypes while SOAP-HLA was used to determine HLA-DPA1 and HLA-DQA1 haplotypes. HLA class I germline genotypes for TCGA NSCLC samples were obtained from the TCGA landscape publication (34) that utilized OptiType (31), while HLA class II germline genotypes were retrieved from the publication of Marty-Pyke et al., 2018 (35).

### HLA loss of heterozygosity

For the primary and validation cohorts, loss of HLA class I germline molecules in the tumors were determined by LOHHLA (36), for which allele specific copy numbers of HLA class I locus were realigned to patient specific reference sequences and corrected by tumor purity and ploidy. Tumor purity and ploidy were assessed in each sample analyzed as described previously (25). In brief, somatic copy number profiles were first determined by mapping reads to exonic and intronic regions (bins) of the genome while correcting for region size, CG content and sequence complexity (37). Next, tumor copy number profiles were compared to a reference panel of matched normal samples to derive copy ratio values. Circular binary segmentation (38) was next applied to copy ratio profiles to determine genomic segment boundaries. Segmental copy ratio values and minor allele frequency of heterozygous single nucleotide polymorphisms (SNPs) overlapping the segment were used to estimate tumor purity and ploidy throughout the genome; all possible combination of tumor purity and ploidy were evaluated for the optimal combination based on maximum likelihood estimation. For HLA class II germline molecule loss in the tumors and all HLA molecule loss in the TCGA samples, the minor allele copy numbers were utilized as previously described (25). Briefly, loss of heterozygosity (LOH) occurred when minor allele copy number of the overlapping genomic region equaled zero. HLA allele specific copy number information of the TCGA samples were obtained from analyses of SNP6 copy number array data on Synapse (https://www.synapse.org/#!Synapse:syn1710464).

### Statistical analysis

Differences between responding and non-responding tumors were evaluated using chi-squared or Fisher’s exact test for categorical variables and the Mann-Whitney (MW) test for continuous variables. Where noted, tumors were classified based on their missense tumor mutational burden (TMB) or IMM load as high or low using the second tertile as a cut-off point. Median point estimate and 95% confidence interval (CI) for overall survival and progression free survival were estimated by the Kaplan-Meier method and survival curves were compared through the nonparametric log-rank test. Univariate Cox proportional hazards regression analysis was used to determine the impact of individual parameters on survival outcomes. All p values were based on two-sided testing and differences were considered significant at p < 0.05.

## Results

### Sex-dependent association between immunogenic mutation load and HLA zygosity

**Figure 1:**
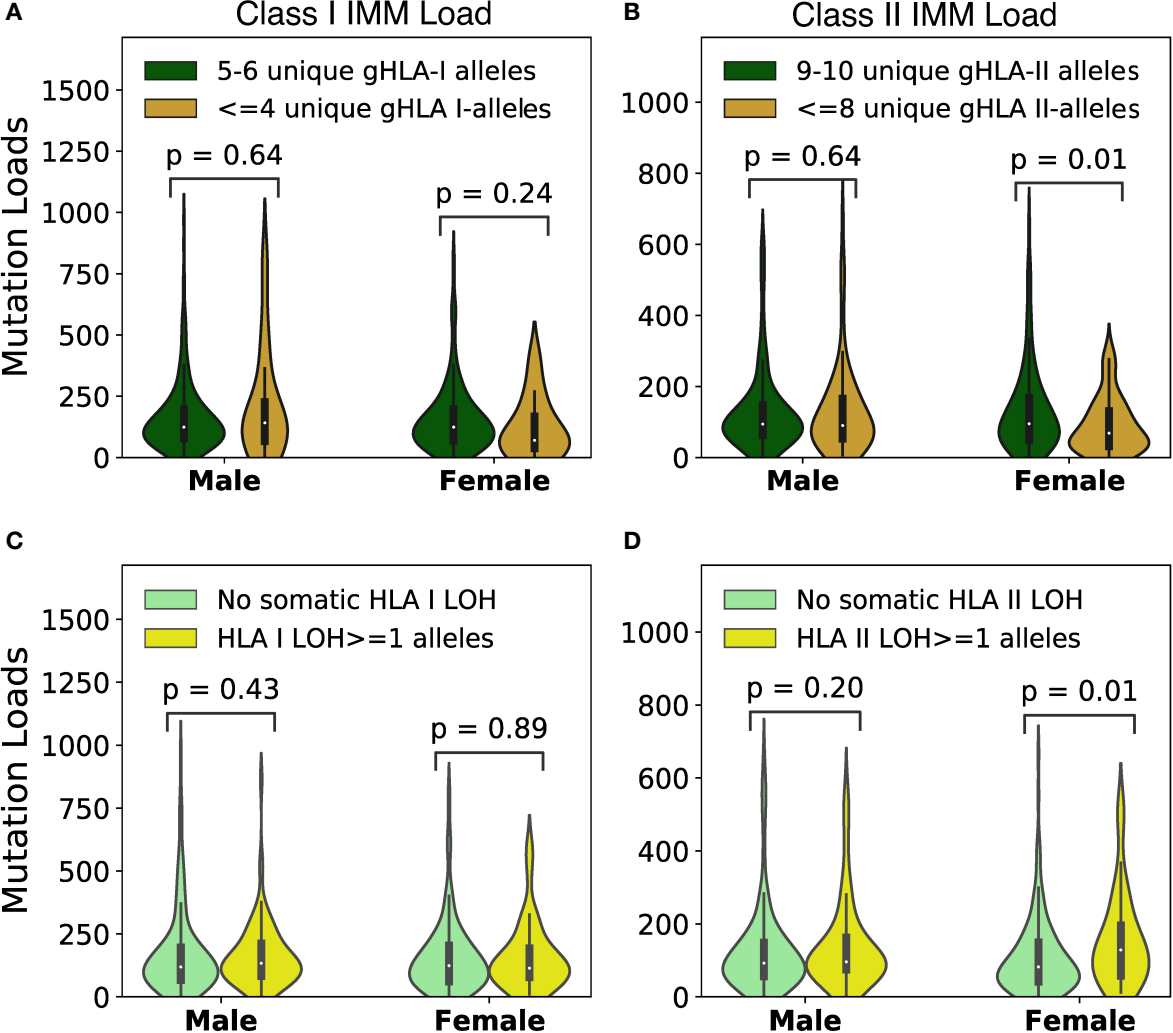
Background immunogenic mutation association with HLA diversity in TCGA-NSCLC cohort. **(A)** No association between class I IMM loads and tumor HLA I diversities was found in either males (MW p=0.64) or females (MW p=0.24). **(B)** Female tumors with high germline HLA II diversity had significantly higher class II IMM loads (MW p=0.01). Male tumors did not show class II IMM load difference between the high germline HLA II diversity and the low germline HLA II diversity groups (MW p=0.64). **(C)** No association between loss of heterozygosity (LOH) of HLA I alleles and class I IMM loads were identified in either males (MW p=0.43) or females (MW p=0.89). **(D)** Female tumors lost ≥1 HLA II alleles had higher class II IMM loads than those with no LOH (MW p=0.01). LOH for HLA II alleles did not associate with class II IMM load difference in male tumors (MW p=0.20).

Cancer immunoediting selects tumor clones that escape immune control throughout tumorigenesis and cancer evolution likely in a sex-specific immune context (39, 40). To investigate sex-based background differences in the immunogenomic landscape of NSCLC tumors independent of therapy, we analyzed HLA zygosity and IMM load in 482 lung cancer tumors from TCGA, including 279 males and 203 females. In males, we did not detect an association between tumor IMM load and germline HLA class I or class II homozygosity (HLA class I MW p=0.64, HLA class II MW p=0.64; **Figures 1A, B**).

Interestingly, and in contrast with previous findings pointing towards a lack of association between HLA germline diversity and TMB (25), the tumor IMM load in females was positively correlated with germline HLA allele diversity, particularly for HLA class II associated IMMs. Among female patients with germline homozygosity in two or more HLA class II alleles, the median predicted class II IMM load was 68.5 as compared to 95 among tumors in females with the most diverse HLA class II repertoire (MW p=0.01; **Figure 1B**). In contrast, the median class II IMM load among males with germline HLA-II homozygosity was 90, as compared to 94 among those with more diverse HLA-II alleles (MW p=0.64). When excluding 45 patients (15 male, 30 female) with activating *EGFR* mutations or *ALK, ROS1*, or *RET* gene fusions, this trend persisted in females, with median tumor class II IMM load of 103 in patients with at least 9 unique germline HLA-II alleles, as compared to 88 in patients with a higher degree of germline HLA homozygosity (MW p=0.06; **Supplementary Figure 1**).

While germline HLA repertoire contributes to tumor-immune recognition and immunoediting, tumors with high neoantigen burdens may escape immune surveillance by somatic loss of heterozygosity of HLA alleles (36). In considering the role of somatic loss of heterozygosity in tumor evolution as a means of immune evasion, we next classified tumors with at least one fewer unique HLA allele in tumor as compared to germline, and observed that loss of heterozygosity at the HLA-II locus was associated with high class II IMM load only in females (MW p=0.014; **Figures 1C, D**). Again, this association persisted when tumors with *EGFR, ALK, ROS1*, or *RET* driver mutations were excluded (MW p=0.02; **Supplementary Figure 1**).

### ICI-treated cohorts description

The primary NSCLC cohort (Anagnostou) included 89 adults with NSCLC treated with ICI therapy, consisting of 46 male and 43 female patients with a median age at ICI treatment initiation of 64 years. The NSCLC validation cohort (Rizvi) included 34 adults with NSCLC treated with pembrolizumab, consisting of 16 male and 18 female patients with a median age of 62.5 years. Demographic, tumor, and treatment information for both cohorts have been previously published (25, 26) and are summarized in **Supplementary Table 1**. Two patients in the primary NSCLC cohort were not evaluable for the durable clinical benefit endpoint.

### HLA class I and II immunogenic mutation load predict ICI response in females

No significant baseline differences were observed between NSCLC tumors of males and females with respect to TMB, class I IMM load, class II IMM load, or mutational smoking signature (for all comparisons MW p≥0.8). Similarly, there was no significant difference in rate of durable clinical benefit (DCB; Fisher’s Exact p=1.0), progression free survival (log-rank p=0.94, HR=1.02, 95% CI 0.62-1.68) or overall survival (log-rank p=0.39, HR=1.31, 95% CI 0.7-2.42) between males and females following ICI therapy.

**Figure 2:**
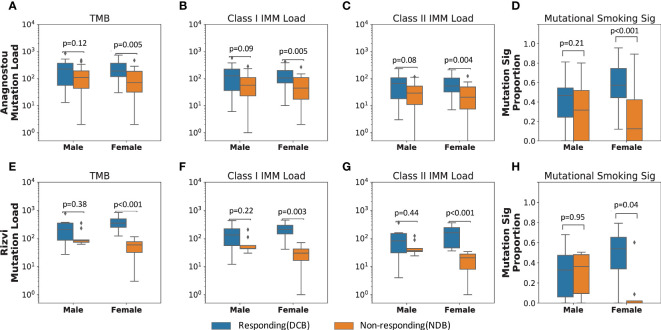
Immunogenic mutation load distinguishes responding from non-responding tumors in females who received immune checkpoint blockade. In the primary NSCLC cohort (Anagnostou), **(A)** female responding tumors harbored significantly higher TMB than female non-responding tumors (MW p=0.005). Similarly, **(B)** Class I and **(C)** class II IMM loads separated female response groups (Class I IMM loads MW p=0.005; Class II IMM loads MW p=0.004), but not male response groups (Class I IMM loads: MW p=0.09; Class II IMM loads: MW p=0.08). **(D)** Smoking mutational signature levels also only differed in female response groups (MW p=0.0006), but not male response groups (MW p=0.21). These results were corroborated in the validation cohort (Rizvi) with immunogenomic features, **(E)** TMB, **(F)** Class I IMM loads, **(G)** Class II IMM loads, and **(H)** mutational smoking signature.

To investigate the difference in immunogenomic features predicting ICI response between males and females, we measured TMB, class I and II IMM load, and mutational smoking signature (Methods) in responding and non-responding tumors by each sex. In female patients, DCB following ICI therapy was significantly associated with higher TMB (MW p=0.005), class I IMM load (MW p=0.005), class II IMM load (MW p=0.004), and mutational smoking signature (MW p=0.0006), while none of these features were significantly different between responding and non-responding tumors in male patients (**Figures 2A–D**).

In order to incorporate the effect of gender- or sex-biased differences like tobacco exposure and prevalence of tumors with driver mutations that typically do not respond to ICI therapy (41, 42), we further stratified the primary cohort by tobacco history and driver gene mutation status. Six tumors harbored activating *EGFR* mutations, while no fusions of *ALK, ROS1*, or *RET* were detected in those with available testing. As expected, none of the six tumors harboring activating *EGFR* alterations (5 female, 1 male) demonstrated DCB in response to ICI therapy. Excluding these six patients from the cohort, the somatic mutational features remained predictive of DCB for NSCLC in females but not in males, including TMB (females MW p=0.03, males MW p=0.13), class I IMM load (females MW p=0.03, males MW p=0.10), class II IMM load (females MW p=0.02, males MW p=0.10), and mutational smoking signature (females MW p=0.005, males MW p=0.26; **Supplementary Table 2**). Among patients with a self-reported history of smoking (35 males, 33 females), the mutational smoking signature trended toward association of ICI response only in tumors in females (median signature contribution of 0.57 in the DCB group versus 0.38 for the NDB group, p=0.06). In contrast, there was no difference in mutational smoking signature in male tumors by therapeutic response (median signature contribution of 0.47 in the DCB group versus median signature contribution of 0.46 in the NDB group, p=0.95).

These findings were corroborated in an independent validation cohort (Rizvi) of 34 patients with ICI-treated NSCLC (26). Among tumors of 18 female patients in the cohort, TMB, class I IMM load, class II IMM load and mutational smoking signature were significantly higher in those attaining DCB as compared to NDB following ICI therapy, including TMB (MW p=0.0009), class I IMM load (MW p=0.003), class II IMM load (MW p=0.0009), and mutational smoking signature (MW p=0.04; **Figures 2E–H**). Among 16 male patients in the cohort, there was no significant difference in these features between responding and non-responding tumors. Similar to the primary cohort, these differences persisted when excluding three patients with tumors harboring *EGFR* alterations (**Supplementary Table 2**).

### Combined HLA zygosity with immunogenic mutation load predicts ICI response in males

Next, we considered the contribution of antigen presentation capacity in addition to IMM load as associated with differential clinical responses to ICI in males and females. We assessed HLA genetic variation as an indicator of neoantigen presentation capacity and its impact on outcomes by sex. Patient germline and tumor HLA haplotypes were classified as high HLA diversity (≥5 unique alleles for HLA class I and ≥9 unique alleles for HLA class II) or low HLA diversity groups (≤4 unique HLA I class alleles or ≤8 HLA class II alleles). There was no significant correlation with germline or tumor HLA zygosity alone and ICI benefit in either sex (**Supplementary Table 3**).

**Figure 3:**
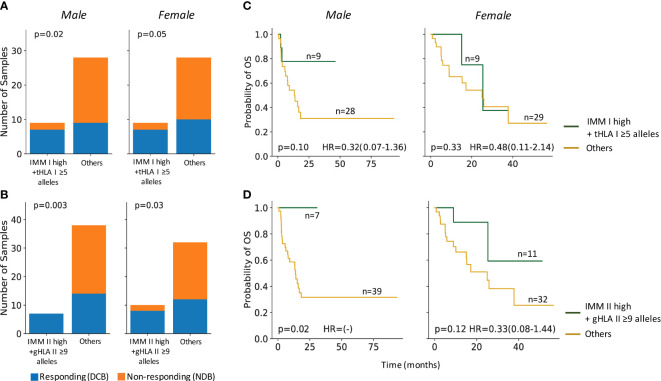
HLA heterozygosity combined with immunogenic mutation loads predicted ICI response and survival in males. **(A)** Male and female tumors with high IMM-I loads and high tumor HLA I diversity (tHLA I ≥5) co-occurred with response to ICI treatments (male Fisher’s Exact p=0.02, female Fisher’s Exact p=0.05). **(B)** Both male and female tumors with high HLA II restricted IMMs and high germline HLA II diversity (gHLA II ≥9) co-occurred with ICI response (male: Fisher’s Exact p=0.003; female: Fisher’s Exact p=0.03). **(C)** Male patients with high tumor IMM-I loads and high tumor HLA I diversity trended towards longer overall survival (OS) (log-rank p=0.1), while their female counterparts did not (log-rank p=0.33). **(D)** Combined high tumor IMM-II loads and high germline HLA II diversity was associated with significantly longer OS among males (log-rank p=0.02) but not females (log-rank p=0.12). Hazard ratio (HR) shown with 95% confidence interval.

Even in tumors with maximal HLA heterozygosity, the potential immunogenicity of mutation-associated neoantigens depends on effective presentation by the patient’s unique inherited MHC repertoire. In contrast to TMB, the predicted IMM load incorporates MHC affinity to estimate the number of neoantigens likely to stimulate an anti-tumor immune response (22). In the primary ICI-treated cohort (Anagnostou), response to ICI correlated with combined high IMM load and high HLA diversity in tumors in both males and females (**Figures 3A, B**). Interestingly, significantly improved OS was only observed in males with high IMM load and high HLA diversity, particularly for HLA class II (log-rank p=0.017; **Figures 3C, D**). This finding in males is consistent with our hypothesis that more highly mutated tumors with intact antigen presentation capacity will benefit from improved outcomes following ICI therapy. In females, HLA diversity did not have a combinatorial effect on IMM load for predicting survival after ICI therapy (**Figures 3C, D**); these findings suggest that alternative mechanisms of immune escape likely contribute to the differential sex-dependent response and outcome following ICI therapy.

## Discussion

Growing evidence points to a sexual dimorphism in response to immunotherapy across multiple tumors that likely arises from differences in immune surveillance in males and females. During tumorigenesis, immune surveillance shapes tumor evolution and selection pressure drives tumor immune escape. We sought to elucidate the immunogenomic features pointing towards mechanisms by which biologic sex influences the tumor-immune interaction that is central to ICI therapy response.

We demonstrate that somatic mutational features, including class I and II predicted IMM load as well as TMB, are associated with durable clinical benefit following ICI therapy in females but not in males. This finding is consistent with recent reports demonstrating that as a predictive biomarker for ICI response, TMB performed better in females than in males, an effect that appears to be independent of smoking signature and oncogenic-driver mutations like *EGFR* (19, 43). Furthermore, in NSCLC, there is no consistent difference in TMB between tumors in males and females, whereas increased TMB is associated with male tumors in melanoma and other tumor types where a sex dimorphism in ICI response has been observed (43–47).

While TMB is a predictive biomarker of response, clinical benefit, and survival after immunotherapy in a variety of cancers, including NSCLC and melanoma (26, 48–52), it is an imperfect biomarker with multiple technical limitations. Utilizing neoantigen predictions to identify somatic mutations with immunogenicity potential has been an effective way to indicate tumor infiltrating leukocyte (TIL) infiltration and survival in multiple solid tumors (22, 23, 34). Combining this approach to estimate tumor immunogenicity with observations of sexual dimorphism in the immune response, we found that higher class I and II IMM load were predictive of ICI response in females with NSCLC.

In line with the predictive role of tumor IMM load in females, mutational smoking signature was also predictive of ICI response only in females. Though NSCLC arising with a history of never-smoking is significantly more common in females (53), there is evidence for a higher impact of tobacco-mediated carcinogenesis in females as compared to males, with increased deleterious effects and higher risk of lung cancer despite a similar degree of tobacco exposure (54, 55). These observations suggest that the interpretation of mutational load as a biomarker for ICI response requires additional context. Similarly, NSCLC with an *EGFR, ALK*, or other non-smoking associated driver alteration is significantly overrepresented among females and is associated with lower TMB and poor ICI response (41, 56, 57). It is likely that observed sex dimorphism in early ICI trials in NSCLC was enhanced by the overrepresentation of never-smokers and select mutation*-*driven tumors among females, however the decreased magnitude of in ICI response in females is also noted in non-smoking associated tumors like melanoma, and in squamous NSCLC not associated with these same oncogenic driver mutations (4, 11, 47).

Antigen presentation capacity in the form of a diverse repertoire of HLA-encoded MHC molecules allows for effective presentation of more tumor-associated neopeptide candidates to stimulate an anti-tumor immune response. As a predictor of response to ICI therapy, HLA-I germline homozygosity in at least one locus and HLA-I loss of heterozygosity have been associated with worse survival and were significantly more negatively prognostic when combined with low TMB (24, 25, 58, 59). We have previously shown that fewer than five unique tumor HLA class I alleles combined with low TMB was associated with lower CD8+ T cell infiltration and was predictive of worse overall survival in this NSCLC cohort, suggesting HLA loss is an adaptive mechanism for immune evasion in tumors with high mutation burden (25). In further examining these patterns by sex, we found that the combination of diverse HLA alleles and high IMM load was predictive of improved survival following ICI therapy in males but not in females, and particularly with respect to HLA class II. This pattern suggests that in females, more immunogenic tumors with high neoantigen burden and intact antigen presentation capacity are under selective pressure to develop alternative mechanisms of immune evasion, favoring immune escape and poor ICI response. In other words, females with already robust tumor-specific immunity will have a lower potential for effect with ICI therapy (i.e. a lower therapeutic index) (60), while males with less baseline anti-tumor response may attain a greater magnitude of therapeutic response.

In further exploring the immunogenomic landscape that evolves in tumors in males and females, we found that maximal HLA class II germline diversity is associated with higher IMM load in females but not in males. This finding indicates that tumors with intact antigen presentation capacity may acquire more novel oncogenic mutations to survive in an environment with more effective anti-tumor immune surveillance. Castro et al. recently demonstrated that females are more likely to accumulate driver mutations early in tumorigenesis that are less effectively presented by their inherited HLA genotype, particularly for HLA class II (40), and thus higher mutation burden may not always reflect increased immunogenicity in females. These data suggest that in an environment of stronger immune selection pressure, tumors in females have developed multiple mechanisms to evade tumor-specific T cell responses, and support our finding that combined high IMM and HLA-II heterozygosity were positive predictors of post-ICI survival in males but not females.

Adaptive tumor alterations in antigen presentation machinery, predominately through selection for loss of heterozygosity of HLA alleles, also promotes immune escape by decreased antigen presentation capacity (36). Loss of heterozygosity at HLA-I loci is common and has been noted in 30-40% of several published NSCLC cohorts (25, 36, 61). Somatic HLA-I loss and low TMB has been associated with poor ICI response (24), and correcting for mutation-associated neoantigens presented by lost HLA alleles identified high TMB tumors with poor response to ICI therapy (61). In analyzing this relationship by sex, we found that HLA-II loss of heterozygosity is also strongly associated with higher IMM load only in tumors in females. Thus, loss of HLA expression as an adaptive mechanism for immune evasion in tumors with high mutational load may be a more prominent influence in females.

The consistent signal in the MHC class II pathway is interesting and highly relevant. While professional antigen presenting cells expressing MHC-II are found in the tumor microenvironment and stimulate CD4+ T cell costimulatory pathways, the intrinsic expression of MHC-II in tumor cells is increasingly recognized as a key pathway in anti-tumor immune responses (62, 63). We recently demonstrated that class II IMM load is associated with ICI response and survival in NSCLC and melanoma (23). A recent prospective randomized phase III trial of combination chemotherapy and anti-PD1 therapy also identified MHC class-II related gene expression as a key factor correlating with survival, especially in low PD-L1 expressing tumors, and was significantly more predictive than MHC-I related gene expression (64). NSCLC tumors in females have increased infiltration of CD4+ T cell populations (19), suggesting that MHC-II driven pathways are central to the anti-tumor immune response in females. Further understanding of the role of the MHC-II and CD4+ T cells will inform not only our understanding of the sex dimorphism in ICI response, but mechanisms of ICI efficacy in all patients.

Future directions will include exploring the multiple sex-based variables at play in cancer development and the immune response, including consideration of cofactors related to additional hormonal, environmental, and genetic influences. Tumors included in each cohort represented a single time point in tumorigenesis, and all ICI treated patients had advanced disease. In light of recent incorporation of immunotherapy in early stage and locally advanced NSCLC (65, 66), it will be important to understand the evolution of these sex-based immunogenomic features and their context in different stages of disease. Similarly, continuing to examine these features with consideration of sex-based differences in response to combination chemotherapy and immunotherapy will be an important next step. Finally, though this analysis considered select driver mutations with known decreased response to ICI and female predominance, a multitude of additional targetable and non-targetable mutations have unclear impact on ICI response, such as *KRAS*, *BRAF*, *MET*, *HER2*, *PI3K*, *PTEN*, and DNA repair genes (42). The complexity of the interaction of these drivers and other co-mutations with sex, smoking exposure, mutational burden and ICI response requires further investigation and emphasizes the need for intricate and individualized modeling for predictive biomarkers.

In summary, the dimorphic activity of the immune system in males and females strongly influences tumorigenesis by immune selection and has important implications in the growing field of cancer immunotherapy. Our data highlight that the interpretation of biomarkers and the mutational landscape of tumors for prediction of ICI therapy response requires consideration of the biological sex context in which the tumor has evolved. Together, the growing clinical and translational data support inclusion of sex as a biological variable in multimodal models of ICI response prediction. This effort requires deeper understanding of the mechanisms underlying sex-driven differences in the tumor-immune interaction, as well as adequate representation of females in prospective clinical trials of immune therapeutic agents.

## Data availability statement

The original contributions presented in the study are included in the article/**Supplementary material**. Further inquiries can be directed to the corresponding author.

## Ethics statement

The studies involving human participants were reviewed and approved by Johns Hopkins Institutional Review Board. The patients/participants provided their written informed consent to participate in this study.

## Author contributions

SS, XS, and VA conceived the study, conducted the data analyses, interpreted the data and wrote the manuscript. NN and AB contributed to data analyses. GP contributed to data collection. KM, VL, JM, JF, BL, DE, CH, JB, PF, RK and JN contributed to data interpretation and manuscript editing. All authors read and approved the final manuscript.

## Funding

This work was supported in part by the US National Institutes of Health grant CA121113 (VA), the Bloomberg-Kimmel Institute for Cancer Immunotherapy (VA, PF), the V Foundation (VA), the LUNGevity Foundation (VA) and the Johns Hopkins Specialized Center for Research Excellence in Sex Differences (U54AG062333; VA, JN).

## Conflict of interest

VA receives research funding to the institution from Astra Zeneca and has received research funding to her institution from Bristol-Myers Squibb in the past 5 years. SS has served in a consulting role for Genentech/Roche. KM has received research funding to the institution from Mirati and Bristol Myers Squibb, and she has served in a consulting role for Amgen, Janssen, Mirati, AstraZeneca and Puma. VL has received research funding to the institution from GlaxoSmithKline, Bristol Myers Squibb, Merck, and SeaGen and he has served in a consulting role for Takeda, SeaGen, Bristol Myers Squibb, AstraZeneca, Guardant Health and Takeda. JM has received research funding to the institution from Merck *via* the Conquer Cancer Young Investigators Award, and has served in a consulting role for MJH Life Sciences, Johnson & Johnson, and Doximity. JF has received research funding to the institution from AstraZeneca, Pfizer, and Bristol Myers Squibb, and has served in a consulting role for Genentech/Roche, Eli Lilly, AstraZeneca, Merck, Takeda, Coherus, Regeneron, and Pfizer. BL has served in a consulting role for AstraZeneca, Daiichi Sankyo, Janssen, Pfizer, Amgen, Takeda, Genentech/Roche, Eli Lilly, Mirati Therapeutics, and Guardant. DE has served in a consulting role for Beyond Spring Pharmaceuticals. CH has received research funding to the institution from AbbVie, Amgen, AstraZeneca, Bristol Myers Squibb, and GlaxoSmithKline, and has served in a consulting role for AbbVie, Amgen, AstraZeneca, Bristol-Myers Squibb, Genentech/Roche, Jannsen and GlaxoSmithKline. JB has received research funding to the institution from AstraZeneca, Bristol Myers Squibb, Genentech/Roche, Merck, RAPT Therapeutics Inc and Revolution Medicines, has served in a consulting role for Amgen, AstraZeneca, BMS, Genentech/Roche, Eli Lilly, GlaxoSmithKline, Merck, Sanofi and Regeneron, and is in the Data and Safety Monitoring Board/Committees of GlaxoSmithKline and Sanofi. PF has received research funding to the institution from AstraZeneca, Bristol Myers Squibb, Novartis, Corvus, Kyowa, and has served as a consultant for Amgen, AstraZeneca, Bristol-Myers Squibb, Daiichi Sankyo, Iteos, Janssen, and as a data safety monitoring board member for Polaris and Flame Therapeutics. JN has received research funding from MSD, AstraZeneca, Bristol Myers Squibb, Amgen, Genentech/Roche, Novartis, has served in a consultant/advisory role for Merck, MSD, AstraZeneca, Bristol Myers Squibb, Genentech/Roche, Pfizer, Takeda, Daiichi Sankyo, Kaleido Biosciences, Amgen and Mirati Therapeutics, and is in the Data and Safety Monitoring Board/Committee of Daiichi Sankyo. Under a license agreement between Genentech and the Johns Hopkins University, XS and RK and the University are entitled to royalty distributions related to technology described in the study discussed in this publication. This arrangement has been reviewed and approved by the Johns Hopkins University in accordance with its conflict of interest policies.

The remaining authors declare that the research was conducted in the absence of any commercial or financial relationships that could be constructed as a potential conflict of interest.

## Publisher’s note

All claims expressed in this article are solely those of the authors and do not necessarily represent those of their affiliated organizations, or those of the publisher, the editors and the reviewers. Any product that may be evaluated in this article, or claim that may be made by its manufacturer, is not guaranteed or endorsed by the publisher.

## Supplementary material

Supplementary Figure 1 Background Immunogenic mutation-HLA diversity associations in TCGA-NSCLC cohort excluding tumors with EGFR, ALK, ROS1, and RET mutations. (A) No association between class I IMM loads and tumor HLA I diversities were found in either males (MW p=0.65) or females (MW p=0.19). (B) Female tumors with high germline HLA II diversity demonstrated trend toward higher class II IMM load (MW p=0.06). Male tumors did not show class II IMM load difference between the high germline HLA II diversity group and the low germline HLA II diversity group (MW p=0.26). (C) No association between loss of heterozygosity (LOH) of HLA I alleles and class I IMM loads were identified in either males (MW p=0.50) or females (MW p=0.94). (D) Female tumors that lost ≥1 HLA II alleles had higher class II IMM loads than those with no LOH (MW p=0.02). LOH for HLA II alleles did not associate with class II IMM load difference in male tumors (MW p=0.36).
