# Supplementary figures and images for: Sex-specific differences in immunogenomic features of response to immune checkpoint blockade
Source: Front Oncol. 2022 Aug 3;12:945798. doi: 10.3389/fonc.2022.945798 (PMC9382103; doi:10.3389/fonc.2022.945798)

**A****Class I IMM Load**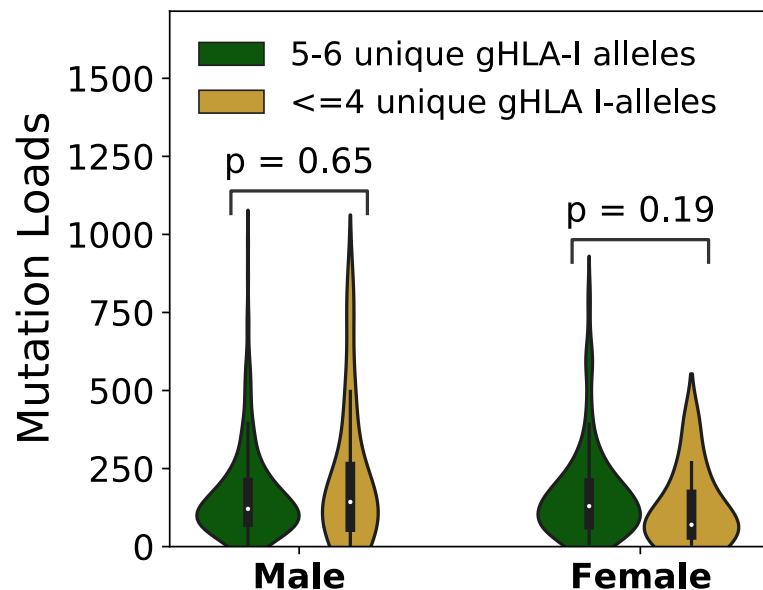**B****Class II IMM Load**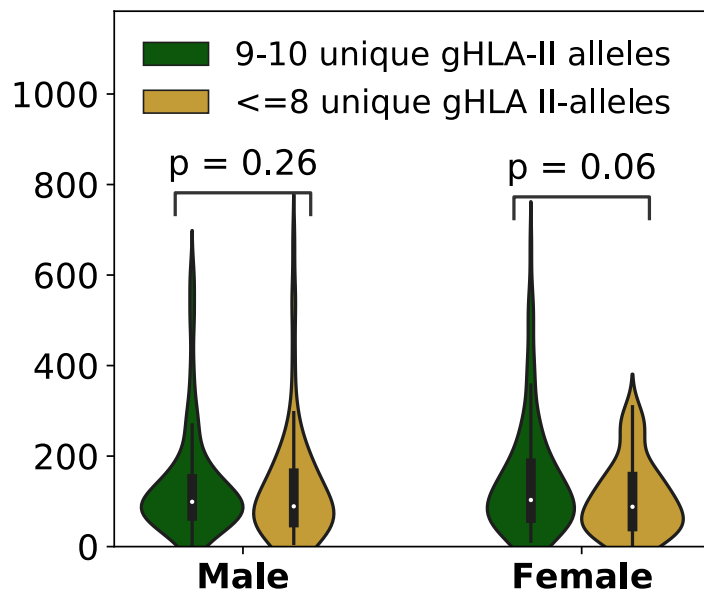**C**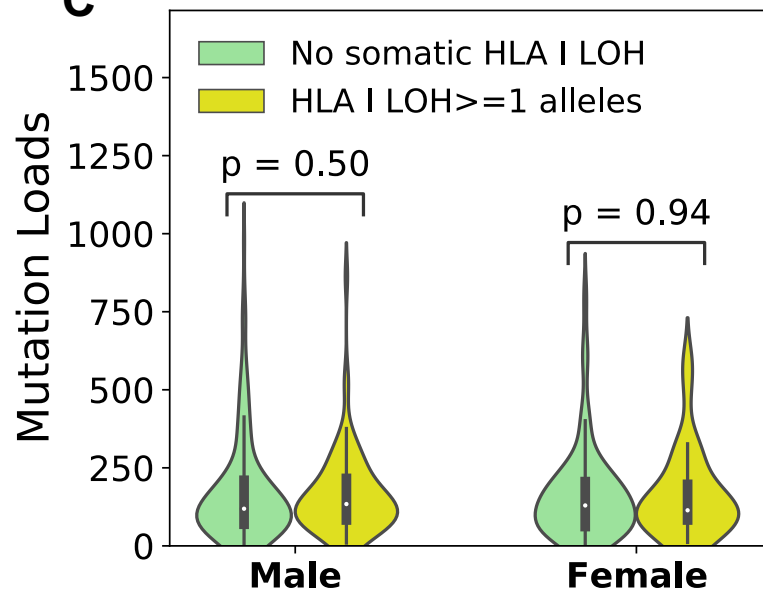**D**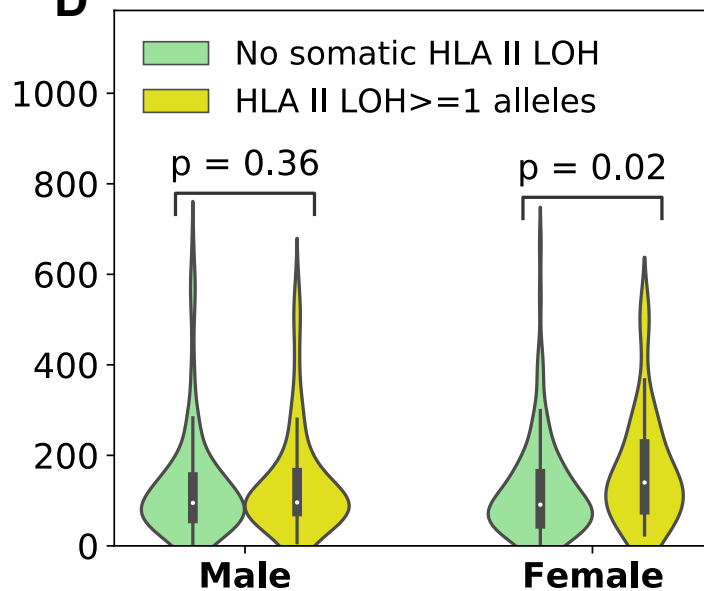

Supplement: Supplementary Figure 1 — Background Immunogenic mutation-HLA diversity associations in TCGA-NSCLC cohort excluding tumors with EGFR, ALK, ROS1, and RET mutations. (A) No association between class I IMM loads and tumor HLA I diversities were found in either males (MW p=0.65) or females (MW p=0.19). (B) Female tumors with high germline HLA II diversity demonstrated trend toward higher class II IMM load (MW p=0.06). Male tumors did not show class II IMM load difference between the high germline HLA II diversity group and the low germline HLA II diversity group (MW p=0.26). (C) No association between loss of heterozygosity (LOH) of HLA I alleles and class I IMM loads were identified in either males (MW p=0.50) or females (MW p=0.94). (D) Female tumors that lost ≥1 HLA II alleles had higher class II IMM loads than those with no LOH (MW p=0.02). LOH for HLA II alleles did not associate with class II IMM load difference in male tumors (MW p=0.36). [file DataSheet_1.pdf]
